# Understanding Reasons for Cancer Disparities in Italy: A Qualitative Study of Barriers and Needs of Cancer Patients and Healthcare Providers

**DOI:** 10.1177/10732748241258589

**Published:** 2024-06-19

**Authors:** Giulia Ferraris, Veronica Coppini, Maria Vittoria Ferrari, Dario Monzani, Roberto Grasso, Gabriella Pravettoni

**Affiliations:** 1 Applied Research Division for Cognitive and Psychological Science, IEO, 9290 European Institute of Oncology IRCCS, Milan, Italy; 2 Laboratory of Behavioral Observation and Research on Human Development, Department of Psychology, Educational Science and Human Movement, University of Palermo, Palermo, Italy; 3 Department of Oncology and Hemato-Oncology, University of Milan, Milan, Italy

**Keywords:** cancer disparities, cancer divide, cancer patients, cancer inequality, digital divide, healthcare providers

## Abstract

**Background:**

The second leading cause of death in Italy is cancer. Substantial disparities persist in the level of care and outcomes for cancer patients across various communities, hospitals, and regions in Italy. While substantial progress has been made in medical research and treatment options, these advancements tend to disproportionately benefit the wealthier, better-educated, and more privileged areas and portions of the population. Therefore, the primary aim of the current study is to explore possible reasons for inequalities in access to and utilisation of care from the perspective of cancer patients, who are recipients of these treatments, and healthcare providers, who are responsible for their administration.

**Methods:**

After being recruited through social media platforms, patients’ organisations, and hospital websites, cancer patients (n = 22) and healthcare providers (n = 16) from various Italian regions participated in online focus group discussions on disparities in access to and provision of care. Video and audio recordings of the interviews were analysed using Thematic analysis.

**Results:**

Among cancer patients, 7 themes were identified, while 6 themes emerged from the healthcare providers highlighting encountered barriers and unmet needs in cancer care. Most of these emerging themes are common to both groups, such as geographical disparities, information deficiencies, and the importance of psycho-oncological support. However, several themes are specific to each group, for instance, cancer patients highlight the financial burden and the poor interactions with healthcare providers, while healthcare providers emphasise the necessity of establishing a stronger specialists’ network and integrating clinical practice and research.

**Conclusion:**

Current findings reveal persistent challenges in cancer care, including long waiting lists and regional disparities, highlighting the need for inclusive healthcare strategies. The value of psycho-oncological support is underscored, as well as the potential of the Internet’s use for informational needs, emphasising the imperative for improved awareness and communication to overcome disparities in cancer care.

## Introduction

Cancer represents a significant global health challenge, with its impact extending beyond the physical aspects of the disease to affect the psychological, social, and economic well-being of individuals and their families.^1^ Despite advances in medical science and healthcare systems, one key aspect of this challenge is the existence of health disparities that are differences in health outcomes or access to healthcare among different populations or groups. Health disparities are often influenced by various social, economic, environmental, and structural factors and play a crucial role in influencing cancer outcomes such as survival rates, disease awareness, quality of life, and the mental well-being of patients and their families. Income, education, geographic location, and ethnicity are some of the most important underlying reasons for disparities in cancer across Italy.^2^ It is vital to address these disparities comprehensively, taking into account various aspects of the healthcare system as well as the needs of cancer patients.^3^ Thus, this study seeks to shed light on these persistent disparities in cancer care in Italy from the perspective of cancer patients and healthcare providers.

The existing literature has emphasised the issue of disparities in cancer outcomes as a matter of critical concern, highlighting several factors possibly contributing to exacerbating such disparities. Firstly, socioeconomic status and ethnic background were found to contribute to disparities in cancer outcomes. Indeed, cancer patients who are at risk of experiencing greater disparities are those with lower socioeconomic status, who are frequently from ethnic minority backgrounds.^4,5^ Secondly, differences in healthcare infrastructure and accessibility, variations in insurance coverage, cultural and language barriers, and disparities in cancer screening and prevention programs can all play a significant role in creating disparities.^6,7^ Furthermore, it is important to recognize the geographic dimension of disparities in cancer, as evidenced by various studies. Such disparities are reflected in certain regions in Italy which expose marked geographic inequalities for example in breast cancer mortality rates.^8,9^ One of the reasons behind geographic disparities in Italy is the decentralisation of the healthcare system, which has determined differences in healthcare access, quality of care, and overall health outcomes across the 20 regions.^10^

Moreover, disparities in cancer outcomes can be further compounded by differences in access to information. Cancer patients frequently require information about prognosis, where to seek specific treatments or examinations, the pros and cons of treatment, and where to find psycho-oncological support.^11^ The main issue resides in the fact that not everyone has equal access to this information, and when these informational needs remain unmet, they are often associated with decreased treatment adherence, increased healthcare costs, anxiety, and depression.^12-14^ The Internet has been cited as the most frequently consulted health information resource.^15,16^ Thus, if digital literacy is well developed, the Internet could be a resource and a means to address these disparities.^17^ However, the Internet is not always accessible to everyone (eg, remote areas and rural centres) and even when online information is available to cancer patients, disparities still persist. Indeed, how people discern sources of cancer information online, the barriers and facilitators of online information-seeking behaviours^18^ and the reasons behind disparities and variations in such behaviours are still relatively unknown.

Lastly, providing psycho-oncological support was found to be a crucial dimension of equitable care. Generally, the risk of emotional distress is notably higher in cancer patients than in the general population.^19^ It is evident that support is needed. However, it is not always accessible to everyone in Italy, with reasons beyond economic factors remaining unclear. In a recent publication,^20^ disparities and barriers in accessing, utilising and providing psycho-oncological support were considered. These inequities, rooted in economic factors, resource allocation, and society awareness levels, highlight a lack of specific budgets for psycho-oncological support in 37% of European countries, with mental health not prioritised in many eastern European nations. Socio-demographic factors such as age, gender, education, income, and residence contribute to an uneven distribution of psycho-oncological support, with disparities among cancer types as well. However, further personal barriers, which may be related to patients and healthcare providers, need to be explored more in depth in order to identify and formulate a strategy to overcome them.

Although many factors were identified as reasons for cancer disparities, it is essential to acknowledge that substantial knowledge gaps persist. While some research has delved into the socio-demographic aspects of these disparities, there is a pressing need to go beyond the surface and explore the underlying reasons comprehensively. This entails not only considering the patients’ perspectives but also that of healthcare providers, which play a crucial role in the provision and improvement of cancer care.^21^ Furthermore, the literature has not adequately addressed the nuanced barriers encountered by both patients and healthcare providers in their respective roles within the complex landscape of cancer care.

Examining disparities in cancer is important for a number of reasons. Firstly, recent data from the European Commission reveal a concerning 2.4% increase in cancer deaths in Italy in 2022 compared to 2020, as reported in the European Cancer Information System.^22^ Additionally, Italy’s cancer incidence rate in 2022 is higher than the EU average, with an estimated 390,700 new cancer diagnoses in 2022, marking an increase of 14,100 cases over 2 years (from 2020).^23^ Despite Italy’s commendable efforts in managing cancer risk factors such as smoking and alcohol consumption, there are evident disparities in participation rates in comprehensive screening programs, primarily influenced by factors such as income and education.^24,25^ These variations underscore the need for targeted interventions to address the root causes of these disparities and ensure equitable access to screening and treatment services across all socioeconomic strata. Secondly, obtaining insights directly from patients and healthcare providers will allow a comprehensive understanding of the challenges and opportunities within the cancer care system. Thirdly, identifying both the barriers and facilitators contributing to disparities in cancer care will enable the development of targeted interventions to address disparities and inequalities.

Thus, the aim of this qualitative study is to explore the underlying reasons and key factors contributing to disparities and inequalities in cancer care, with a specific emphasis on barriers and needs related to diagnosis, treatment, psycho-oncological support, information-seeking behaviours, and decision-making processes of cancer patients and healthcare providers. By addressing these issues comprehensively, we aim to contribute to a more equitable, patient-centred, and effective cancer care system, ultimately improving the lives of those affected by cancer. The present study is part of a wider project named “Cancer Care BEACON'' that will create a decision support tool that aims to assist stakeholders (eg, cancer patients, healthcare providers, researchers and policy-makers) in making informed decisions related to cancer care. For example, these tools will include resources to empower patients in making informed decisions about the most suitable facilities for their cancer needs, to support healthcare providers with education materials and guidelines, to assist researchers in identifying appropriate hospitals for conducting future clinical trials, and to serve policy-makers by collecting information on policy initiatives and reports crucial for informed decision-making in cancer healthcare policy. The BEACON consortium comprises a team of psychologists, oncologists, data scientists and policymakers from different EU countries: European Institute of Oncology (IEO; Italy), SporeData (SD; Estonia), University of Palermo (UNIPA; Italy), the European Alliance for Personalised Medicine (EAPM; Slovenia), and the Klinicki bolnicki centar sestre Milosrdnice ustanova (SMUHC; Croatia).

## Methods

### Participants

Participants were recruited between April 2023 and November 2023. Eligibility criteria were carefully defined. Cancer patients who were 18 years of age or older, those who had received a cancer diagnosis at any point in their lifetime, or those who were actively serving as caregivers for individuals with a cancer diagnosis were included. Concerning the healthcare providers, participants specialised in cancer diseases (ie, medical oncologists, surgeons, clinical oncologists, psycho-oncologists, palliative care specialists, physical and rehabilitative medicine specialists) and working in an Italian hospital, centre or healthcare facility were included. Additionally, all participants from both groups were required to be fluent in Italian and have reliable Internet access to facilitate their participation in online focus group discussions. Family members or caregivers of cancer patients were included, as well as cancer survivors, in order to provide a wider perspective of the experience of a cancer patient. Exclusion criteria were restricted to individuals without Internet access and those who had not received a cancer diagnosis themselves or were not caregivers to cancer patients. Recruitment was conducted through various social media platforms, such as Facebook and Twitter, hospital websites and thanks to the collaboration of patients’ organisations. A total of 40 patients and 25 healthcare providers and researchers were invited to participate in the discussions.

### Procedure

The reporting of this study conforms to COREQ guidelines^26^ [see supplementary file 1]. This study was part of the wider BEACON project; the full details of the BEACON study, including objectives and methodologies, are comprehensively documented in the protocol study.^27^ The current study specifically focuses on gathering qualitative insights from cancer patients and healthcare providers regarding disparities in cancer care provision or reception. After obtaining ethical approval from the Bioethics Committee of the University of Palermo, all participants were required to provide informed consent, which was obtained through electronic means before the focus group discussions were held. To organise the online discussions of around 50 minutes each, participants were grouped into 5 separate sessions for cancer patients and 4 sessions for the healthcare providers, as data saturation was reached. Each session was moderated by 2 trained psychologists from the who are also co-authors of the present study. Data saturation is defined as “the point at which no new information or themes are observed in the data”. To determine data saturation, we evaluated information redundancy accompanied by a diminishing emergence of new themes in focus group discussions.^28^

During the online discussion, GF and VC explained the rules of the focus group discussion, provided more detailed information about BEACON, and guided the group ensuring that all the participants had the opportunity to share their experiences, perspectives, and insights regarding the disparities they have perceived or known of. During the interview field notes of the discussion were taken along with audio-video recordings. Additionally, the online format allowed for geographical diversity, not only in terms of ruralness of residence but also of a variety of Italian regions potentially allowing more heterogeneous responses.

### Data Analysis

The analytical process in this study adhered to the key stages of qualitative thematic analysis, as outlined by Clarke and Braun.^29^ Data analysis was conducted by two female trained research psychologists (GF, PhD; VC, MSc) independently to ensure robust and comprehensive results. A bottom-up qualitative thematic analysis approach was employed, emphasising the emergence of themes from the data rather than imposing a pre-existing coding framework. This approach allowed for a deep exploration of participants’ experiences and perspectives. In the initial phase, after the transcription of the audio and video recordings of the focus group sessions, and after anonymisation processes, two authors (GF and VC) thoroughly read each text multiple times to become well-acquainted with the content. During the second phase, authors carried out an initial coding of the data, utilising codes to identify segments within the textual reports, based on their semantic content. This process facilitated the systematic organisation of the data. In the third phase, the various codes were aggregated into sub-themes and potential main themes with specific attention to identifying barriers and facilitators as well as the preferences and needs of participants. This step allowed for a deeper exploration of all the nuances within the data. Any discrepancies in coding and theme identification were resolved through discussions among the authors to ensure inter-rater reliability and consistency in the analysis process. In the fourth phase, the emerging themes were further developed and reviewed for coherence and relevance to the research question. This step aimed at refining the themes, assuring they accurately reflected the data. In the final phase, the themes were labelled, accompanied by explicit definitions for each theme to add clarity and transparency to the analysis. To further validate the emerged themes, an author who was not involved in the earlier phases of analysis (DM) scrutinised the entire process and the identified themes. External validation adds credibility and robustness to the emerging themes.^29^ The focus group interview guides comprised different open questions aimed at exploring cancer patients’ difficulties in accessing care, potential delays and reasons for delays in diagnosis or treatment decisions, barriers to patients’ information-seeking behaviours and decision-making processes, unmet needs and challenges in accessing psychological support. For healthcare providers, the interview guide aimed at exploring the ways they see integration between clinical practice and innovative research, difficulties in communicating with certain patients, and challenges in participating in multidisciplinary teams. The whole interview guide can be found in the study protocol paper.^27^

## Results

### Patients’ Characteristics

Five focus group discussions were held with 22 cancer patients out of the 40 invited. The age of the participants ranged from 45 to 70 years (M = 56.4; SD = 8.4), and 81.8% of the participants were female. The majority (54.5) of the participants had a diagnosis of breast cancer followed by gastrointestinal cancer (22.7%). The participants resided in 10 different Italian regions, the majority were from Sardinia (22.7%), Lombardy (18.2%) and Piedmont (18.2%). The sample was roughly divided into Northern (54.5%), Central (4.5%) and Southern (41%) regions in order to give a clearer picture of its distribution across the Italian peninsula. Additionally, almost half of the patients resided in cities or central areas (45.5%), a wide percentage (40.9%) in suburban areas and a small number of patients were living in rural areas (13.6%). Further information regarding the patient’s health status is presented in Table 1.

**Table 1.** Italian Patients’ Health Status.

Health Status	Patients	
	*n*	%
Latest Diagnosis		
Breast	12	54.5
Gastrointestinal	5	22.7
Lung	2	9.1
Ovary	2	9.1
Prostate	1	4.5
Time since diagnosis		
year	5	22.7
1-3 years	8	36.4
3-5 years	3	13.6
>5 years	6	27.3
Previous diagnosis		
No	16	72.7
Breast	4	18.2
Pancreas	1	4.5
Fibroelastoma dorsum	1	4.5
Comorbid health diseases		
No	6	27.3
Chronic disease	2	9.1
Mental health problems	1	4.5
Asthma	1	4.5
Physical impairment or disability	8	36.4
Joints problems	1	4.5
Multiple pathologies	3	13.6
Care and support		
No	13	59.1
Spouse/Partner	6	27.3
Son	1	4.5
Parent	1	4.5
Friends/Family	1	4.5

### Healthcare Providers’ Characteristics

Four focus group discussions were held with 16 healthcare providers, out of the 25 invited, 10 were medical oncologists with 2 of them also surgical oncologists, 4 were psycho-oncologists and 2 were radiation oncologists. Their age ranged between 31 and 65 years (M = 44.9; SD = 8.8) and 56.2% were female. Healthcare providers worked among hospitals and cancer centres across 4 different Italian regions, the majority in Lombardy (56.2%), a few in Piedmont (18.8%) and Sicily (25%) and the last 2 participants worked in Lazio and in the Marche region. The sample was divided into Northern (62.5%), Southern (25%), and Central regions (12.5%). Additional information regarding the healthcare providers’ expertise is provided in the supplementary file 2, while further socio-demographic information regarding both patients and healthcare providers is outlined in Table 2.

**Table 2.** Sociodemographic Characteristics of Italian Patients and Healthcare Providers.

Sociodemographic Characteristics	Patients		Healthcare Providers	
	*n*	%	*n*	%
Gender				
Female	18	81.8	9	56.2
Male	4	18.2	7	43.8
Marital status				
Single	5	22.7	3	18.8
Married	12	54.5	9	56.2
Partnered	3	13.6	3	18.8
Divorced	2	9.1	1	6.2
Widowed	0	0	0	0
Other	0	0	0	0
Ethnicity				
Western european and central mediterranean	21	95.5	15	93.8
Rather not answer	1	4.5	1	6.2
Highest educational level				
Elementary schoolMiddle school				
High school	12	54.5		
University or postgraduate degree	9	40.9	16	100
Other	1	4.5		
Employment				
Unemployed	11	50		
Employed full-time	9	40.9	16	100
Employed part-time	2	9.1		
Ruralness of residence				
City/Central area	10	45.5	N/A	
Suburban area	9	40.9		
Rural area	3	13.6		
Geographical area				
Northern Italy	12	54.5	12	62.5
Central Italy	1	4.5	2	12.5
Southern Italy	9	40.9	4	25
Monthly income				
< 1100 €	2	9.1	N/A	
> 1100 < 1600 €	3	13.6		
> 1600 < 2500 €	4	18.2		
> 2500 €	1	4.5		
Rather not answer	1	4.5		

### Patients’ Themes

The analysis uncovered 7 distinct themes. In each discussion, prior to diving into the topics outlined in our guide, participants naturally began by sharing their personal experiences of having received a cancer diagnosis. In the subsequent paragraphs, each of these themes will be explored. Subthemes will be described and examples of quotes extracted from the transcripts will be reported in more detail in Table 3

**Table 3.** Themes, Sub-themes and Example Quotes From Italian Patients.

Themes and Subthemes	Example Quote
**Long waiting lists and bureaucracy**	
Excessively long waiting lists	“For follow-up exams, the waiting times are not just extremely long. More than that! some waits extend for years!”
Feeling of abandonment and psychological burden	“The biggest difficulty for me, which I found very negative and psychologically draining, was the very long waiting lists, everywhere, for every little thing, even for a piece of information.”
Bureaucracy and complications within the public healthcare system	“The hitches…the oncologist prescribed the appointment for breast reconstruction, but I had already undergone that visit, and she said-I am sorry, but you are no longer on the list-and I had to go through the process of booking the right exam again.”
**Geographical and regional disparities in healthcare resources**	
Lack of facilities, techniques and equipment in central and southern regions	“I live in Sardinia, a part of Italy that we realise when we are sick that we are really abandoned. They told me that they do not even do that kind of surgery in Cagliari, it was too complex an operation to be performed there.”
Non-existent or inadequate healthcare services in smaller cities and rural areas	“This is because in Olbia the necessary machinery for radiation therapy did not exist at that time, and in Nuoro there were endless waiting lists, so I was at risk for not receiving the therapy in the advised period of time.”
Physical limitations and lack of infrastructure preventing access to facilities that are distant from the place of residence	“I started going to physical therapy two months ago. Unfortunately, I am now limited in driving, so I have to rely on buses. However, the issue is that I am physically weak, and my legs feel heavy, so I stopped going.”
**The need for a more accessible and available ** **psycho-** **oncological** ** support**	
Lack of information and awareness about the availability of and access to the support services	“I did not know that this service existed because no 1 had told me, but I would have needed it. I discovered it thanks to a patient association.”
Lack of dedicated spaces and resources	“There is the support service in Trieste. However, in reality, it cannot handle all the patients because there is only one psychologist in the hospital so, practically, this service does not exist.”
Neglect of psychological aspects by medical staff and lack of referrals	“Not enough attention is given to the psychological aspect, how do doctors assess when giving diagnoses whether a person is worthy of a psychologist or not? I needed the support and it was not given to me.”
Long waiting lists	“I started private psychological therapy because with the public healthcare system I would have had to wait four-five months…but I needed it right away.”
**Lack of empathetic patient-provider interactions and effective communication among specialists**	
Superficial and detached attitude while talking to patients	“The biggest problem I had was with the oncologist, I found him so lacking in communication that I even felt uncomfortable, I did not know what to do...If you could change oncologist...He did not interact well with the patient and explained absolutely nothing to me, I was in total darkness."
Lack of effective communication among specialists	“There was an error on the part of the national institute of cancer because they did not think to have it done, but it was necessary…the doctors…they do not talk to each other and it affects us patients!”
Misinformation and wrong referrals from the general practitioner	“My general practitioner did not help me at all, I had to insist on myself. I asked him about disability benefits, the 104 [Italian law providing special protection, services and assistance to people with disabilities], and he said - What is the point? Just take sick leave from work.- and someone who does not know about this law might not even ask.”
**The financial burden that comes with a cancer diagnosis**	
Travel costs	“Getting to Milan was gruelling, it involved a considerable expense. A series of financial costs, as well as stress and effort that I would have avoided if I could have received treatment in Cagliari.”
Privatisation of healthcare and political issues, need for funds and investments	“Not everyone has the money to pay and go privately…, but in Italy there is a move towards private healthcare, either due to lack of investment or political will… But healthcare should be affordable for everyone and equal also at regional level.”
Inability to get treatment in other European countries without private insurance	“Why cannot I seek an innovative treatment, for example in The Netherlands, without private insurance? It is not fair for a cancer patient!”
**Insufficient and inaccessible information**	
Insufficient or unhelpful information	“In my imagination the role of the family doctor was supposed to be this, if nothing else at least to give general indications of the path to be followed and the various stages. Instead, it is very narrow and they give you one piece of information after another at each step, all broken down and very restricted”
Ambivalence on the use of Internet	“The Internet absolutely not, I have never looked up specific things about the disease because I do not like it, I prefer to rely on what the doctor tells me.” “Everything I learned, I have learned through the Internet. The Cuneo cancer association does absolutely nothing and does not provide any information either.”
Unreliable or unclear information on the Internet	“We do not refer to Google; this is a big problem in my opinion nowadays. If someone has the ability to distinguish truly scientific information from all the web hoaxes, then yes, but in patients, it is difficult.”
**Scarce involvement in ** **decision-** **making** ** processes**	
No consideration of treatment toxicities from healthcare providers	“The oncologist who was taking care of me wanted me to undergo a treatment, but I had reported issues with that therapy…”
Seeking for a second opinion	“I wanted to decide and so I asked for a second opinion, I was advised to do the draining and decided to do it.”
Strictness of treatment protocols	“The protocols are rigid, they do not allow for much change, so even if the oncologist would like to compromise a little, the instruments do not allow for that much…and once you exit the protocol you cannot access it again that easily…bureaucracy complicates everything.”

#### Long Waiting Lists and Bureaucracy

The majority of participants expressed frustration with excessively long waiting lists both for exams and procedures, as well as the reception of useful and necessary information, indicating a need for reduced waiting times to enhance patient access and well-being. Frequently, patients stated that they understood that the Italian medical staff is overloaded with work and patients, nonetheless, they felt abandoned and psychologically burdened by the exceedingly prolonged waiting lists. Moreover, bureaucracy and complication in the public healthcare system were also significant barriers, for more than half of the cancer patients. This includes difficulties in obtaining accurate prescriptions of appointments and medical exams, in maintaining one’s own position in the waiting list, avoiding further delays in diagnosis and treatment. Additionally, rigidity in the sequence of steps, lack of uniformity among regional protocols and subdivision of responsibilities among different professionals, generate difficulties in comprehending the pathways between appointments and exemption possibilities. Specifically, patients do not know whom to turn to for this information and what rights they are entitled to, sometimes discovering them by chance. For instance, one patient complained about the lack of necessary and practical information: “*I did not know anything about exemptions. I did not know that Sardinia reimbursed expenses for meals and travel costs. I discovered it after months and months during which I had to cover all the expenses myself”*.

On the other side, facilitators in accessing care included having personal connections with healthcare professionals. These relationships foster trust and communication between patients and medical staff. Furthermore, participants appreciated the ease of booking appointments and the efficiency of medical visits and test results, underscoring the importance of a streamlined healthcare system.

#### Geographical and Regional Disparities in Healthcare Resources

Geographical and regional disparities emerged as a significant concern for the majority of the participants. Indeed, participants reported that the healthcare resources are not equally distributed across different regions. Particularly, cancer patients living in less developed or remote areas, like the islands (ie, Sardinia and Sicily), often face challenges in accessing adequate medical facilities and essential equipment for treating specific types of tumours. The most prevalent barrier, mentioned by the vast majority of participants, was the lack of facilities, training, resources, techniques, and equipment in various hospitals and cancer centres in central and southern regions. Similarly, participants from smaller cities and rural areas experienced greater difficulties in obtaining adequate care compared to those living in larger cities. For example, one patient from a southern region clearly emphasised the issue of regional disparities: “*There should be no regional healthcare, but we should all have the possibility of being treated as close to home*”.

#### The Need for More Accessible and Available Psycho-Oncological Support

The majority of participants agreed on the utility and the value of psychological support in the oncological field, with 57.9% of participants having a positive opinion of the usefulness of psycho-oncological support, while 10.5% held a neutral or negative view and the rest did not express an opinion on the matter. However, psycho-oncological support is often lacking due to misinformation and the limited availability of resources and dedicated spaces. Further, many participants expressed frustration with excessively long waiting lists, which hindered their access to psycho-oncological services. Additionally, patients reported to be unaware of the existence of such mental health services. This was the case of one participant, living on an island, who was unaware of the possibility of psycho-oncological support: *“I think the support of a psycho-oncologist is very important for the patient and the family, unfortunately in Olbia, there is no psycho-oncologist so who can you turn to?”*. In these cases, the support offered through associations, online communities, and the exchange of experiences with fellow patients constituted a valid means of support for the majority of the cancer patients who took part in the current study. In general, patients require comprehensive knowledge of the availability of psychosocial resources to meet their needs and preferences. The utility and importance of patients’ groups could be grasped from the following quote: “*I gained great benefit from sharing notes of lived life, which have certainly alleviated my situation. Sharing is a powerful weapon. It helped me through hard times*”.

Lastly, some patients who experienced a lack of empathy from medical staff, highlighted the tendency of medical professionals to neglect the psychological well-being of the patients and treat them as “numbers” and not actual people.

#### Lack of Empathetic Patient-Provider Interactions and of Effective Communication Among Specialists

Another recurring theme that emerged during the focus group discussions, concerned the interactions between patients and healthcare providers. Many of the participants expressed their frustration with what they perceived as a superficial and detached attitude expressed by their doctors. They shared experiences of feeling lost and uncomfortable during their interactions with oncologists, describing a lack of effective communication that left them disoriented. Furthermore, patients recounted instances of misjudgement and misinformation from both oncologists and, especially, general practitioners. General practitioners did not prove useful for most patients, as well as not knowing the rights of the cancer patient, or not being able to refer the patient to the correct specialist. Many patients pointed out the lack of general practitioners, especially in small villages, and their difficulty due to the huge number of patients per doctor. Overall, patients expressed frustration at the healthcare system’s inability to provide them with adequate medical support, assistance with bureaucratic procedures, and guidance on their rights as cancer patients. These interactions emerged as significant barriers to their cancer journey, with a substantial number of patients (45.5%) highlighting dissatisfaction with their general practitioners and an even higher percentage (55%) expressing a desire for more attention and availability from their medical providers.

Moreover, collaboration among specialists was highlighted by a few patients, suggesting that a more cohesive approach among healthcare professionals could lead to improved patient outcomes. Additionally, the neglect of therapy-related side effects was identified as an area in need of attentive consideration. However, the most significant facilitator, as emphasised by half of the participants, was the quality of the interaction and relationship between patients and their medical providers. Patients wished for genuine care, empathy, and open communication from their healthcare team. Furthermore, they stressed the importance of having medical professionals who were readily available to provide support and information throughout their cancer experience, as reported by one participant: “*I am lucky because I have a very good general practitioner, he has a 360-degree view of the patient and tells you which specialist to go to depending on what needs to be done*”.

#### The Financial Burden that Comes with a Cancer Diagnosis

The financial aspects of dealing with cancer emerged as a prominent theme during the discussions with cancer patients. Patients voiced their concerns about the economic burdens associated with their illness, particularly in terms of the costs related to travel between regions and from provincial towns to larger cities for treatment. This geographical disparity created a significant financial strain for many individuals and their families. Furthermore, the extended waiting lists for public healthcare services led to another challenge, compelling patients to seek private healthcare alternatives, even for essential psychological support. This private healthcare pathway, though providing quicker access to care, came with substantial costs that added to the financial burden already borne by patients and their loved ones. The financial challenges associated with cancer care were identified as significant barriers to patients’ well-being. Nearly one-third of the participants highlighted the economic costs as a substantial obstacle. The broader issues of healthcare privatisation, political factors, and the need for increased funding and investment were also recognised as barriers by the majority of the participants.

Some patients expressed frustration over the inability to seek treatment in other European countries without private insurance, further compounding their financial concerns. Conversely, the facilitators in addressing these financial challenges were less prominent in the discussions. Only a small number of patients mentioned the importance of financial availability and having insurance as a potential facilitator.

#### Insufficient and Inaccessible Information

The participants highlighted the critical importance of having access to clear and comprehensible information about available treatment options when dealing with cancer. They expressed their frustration with the often insufficient or unhelpful information provided by doctors, which led them to online searches (ie, the Internet) for more comprehensive insights. However, this reliance on the Internet is not always possible and some patients reported to be aware of the existence of unreliable sources online. Access to reliable information emerged as a great need and a potential facilitator in enhancing knowledge about the disease. Furthermore, the Internet served as a valuable tool for connecting with fellow patients and relevant associations through platforms like Facebook groups and pages.

The barriers to obtaining essential information were multifaceted, with more than half of the participants identifying the lack of awareness about reliable and useful information sources as a significant challenge. Additionally, the shortage of informative and empathetic doctors was highlighted by the participants as a substantial barrier. On the other hand, several facilitators emerged to address these challenges. Almost half of the participants emphasised the value of doctors who are thorough and available in providing the needed information. Some patients expressed trust in medical professionals over online sources, choosing not to turn to “Dr Google.” Word of mouth and personal connections were also considered helpful in gaining knowledge about the disease. Associations dedicated to cancer information and the exchange and discussion with other patients played crucial roles in enhancing understanding. Despite initial reservations, the Internet was recognised as both a facilitator and a barrier. Some patients found it to be a useful source of information, while others mentioned using it to connect with resources and other patients. Additionally, books and scientific literature were mentioned as sources of information by a minority of participants.

#### Scarce Involvement in Decision-making Processes

The decision-making process within the healthcare context is crucial for patients, and it can be influenced by various factors, as indicated by current findings. The main significant barrier, reported by at least half of the participants, was the limited possibility of actively participating in the decision-making regarding cancer treatment. Most participants stated that healthcare providers made decisions on their behalf and they had to accept it as it was, even when they had concerns about the possible toxicity or had experienced severe side effects. This has led most of the participants to seek a second opinion in another hospital or through a private consultation to be able to express their feelings and concerns about the suggested treatment. A substantial portion of the patients mentioned the strictness of treatment protocols being a rigid barrier to the possibility of active involvement in treatment decisions, reducing the flexibility of available options.

Some participants, however, were able to negotiate changes in their treatment plans based on their health conditions and preferences by taking a firm position and refusing the healthcare providers’ proposals. In addition, several patients requested specific genetic tests as they knew from official sources, or word of mouth, that were crucial to take before surgery or treatment and as a preventive action. They expressed the importance of being the protagonist in their illness and making decisions aligned with their preferences, even if it meant going against medical advice. A stubborn attitude of the patients or their caregivers, and advocacy for informed personal choices have worked as facilitators in making treatment decisions. For example: *“I decided not to take the medication. Knowing the side effects of chemotherapy, I said -I* *am not doing chemo, solve my problem differently- and they listened to me, and they accepted.”*

Another facilitator that emerged was the advice and recommendations from doctors and nurses when they were clear and collaborative, but mostly when the patients had the feeling that the suggestions were given with their best interest in mind and with openness to questions and confrontation. Again, the empowerment of patients to make informed decisions facilitated the patient's experiences in decision-making processes. Paradoxically, the absence of active participation in decision-making was seen as a facilitator for some participants. In certain situations, patients may prefer to place their trust entirely in the expertise of their healthcare providers, allowing them to make decisions on their behalf Table 4.

**Table 4.** Patients’ Informational Needs.

Type or area of the needed information
Which exams and visits to undergo
Where to go for exams and visits
Whom to refer to/contact to obtain medications/visit or exam appointments/treatment/support
What are the possible and alternative treatment options
The oncology patient’s rights
Which are the facilities with shorter waiting lists
Where are the facilities with shorter waiting lists
The correct diet or lifestyle to adopt coherently to the personal health status
Which are the best facilities and/or specialists
Whether there are opportunities to participate in clinical studies and how to apply

*Note.* The current table reports the informational needs that emerged from the focus group transcripts.

### Healthcare providers’ themes

The analysis of the focus group discussions with healthcare providers resulted in 6 main themes that will be explored below. Subthemes will be described and examples of quotes extracted from the transcripts will be reported in more detail in Table 5.

**Table 5.** Themes, Sub-themes and Quotes Examples From Italian Healthcare Providers.

Themes and Subthemes	Example Quote
**Excessively long waiting lists and clinical workload**	
Lack of dedicated spaces and resources in the public healthcare system	“Once you occupy that space, that space is taken. So, in my opinion, a major limitation we have is a structural limitation…”
Overwhelming burden and strain on healthcare providers	“For us the big problem is the lack of personnel…fatigue with such small numbers of physicians in the hospital setting…”
**Geographical and regional disparities in healthcare resources**	
Lack of diagnostic tools impacting survival	“Tragically, by the time the diagnosis was confirmed, the patient had already passed away. This delay is considered unacceptable and highlights the critical need for expedited diagnostic processes in cancer care.”
Disparities in screening awareness and innovative treatment availability	“The types of machines that are used to monitor, for example, the progress of treatment as dr X was saying, rather than to understand the state of the art with respect to the pathology are different depending on the economic state of well-being of the facility where the examinations are performed.”
**The need for greater involvement of psycho-oncologists**	
Lack of specialized personnel	“There are no trained specialists, yes they may be psychologists, but they are not trained to support cancer patients…so there is no actual service.”
Challenges in patients’ acceptance	“The most significant challenges arise in situations where family members recognize the need for psychological support, but patients adamantly refuse it, creating a common and intricate scenario…it becomes really challenging. Overcoming this resistance demands a nuanced approach, addressing both patient and family concerns, making it a complex yet vital aspect of cancer care.”
Refrain from referrals due to lack of personnel and dedicated spaces	“We usually refer the patient to the support only if we see that there is significant distress, or if the patient requests it…you know because there is no space and time for everybody, it will not be possible…”
The psycho-oncologist as a facilitator of the patient-provider communication	“To have the psychologist at the communication of the diagnosis would be great, but impossible with the resources we have…Unfortunately, the psychologist is outside; they are at the entrance, and I am on the opposite side of the hospital.”
**Difficulty in involving patients in shared decision-making**	
Significant time constraints to the possibility of involving patients in decision-making	“Most of the time, the decision cannot be shared so much because a timely intervention determines survival; the decision is what needs to be done, that is unless there are very strong objections, on certain things… you try to meet halfway, though...you cannot wait because it is a matter of life or death.”
Strictness of clinical protocols	“Yes, as far as possible we are all open to compromise, on toxicity and… But I mean all that margin… I mean the first line in the head-neck is that 1 there, in the sense of… what we have to discuss...that is the best protocol!”
Lack of time for an adequate education of the patient	“In everyday life you realise that shared decision making just is not possible because there is always that information gap that you can take hours to explain to the patient what his illness is and what the treatment options are and various things, but it will still be an information gap that a little bit is because there is a diversity of skills and certainly of education and training…”
**The need for patients’ empowerment**	
Limited availability of information	“...The problem is how the patient enters the pathway… it is often very difficult, most patients don’t know how to navigate the system, there is a lack of guidance. This lack of structured information often leads to delays in diagnosis, making it crucial for us to find ways to simplify the process and provide clearer instructions for patients to access the necessary medical care. The patient needs to be empowered how you say.”
Inadequacy of the general practitioners	“General practitioners have a lot of work, and they are the problem… The patient who already comes to the oncologist is already very advanced… The problem is getting there… patients still arrive having paid 800 for a gastroscopy… Because they could not find a way to get it done.”
Lack of patients’ associations in some regions or cities	“In Piedmont, patient associations do not seem to gain much ground…I do not know why, I do not know really…in my opinion, however, they can be a good informational vehicle for patients, a source they would willingly listen to, but in Piedmont they do not find much space.”
**Limitation in the integration of clinical practice and research activities**	
Excessive workloads and lack of time	“We cannot manage it… In the sense that at least 80-20, but even all the research and lecture preparation is done at home, when you are not at the hospital, because otherwise, you cannot manage it. We have few specialized doctors even though it isa university hospital, things might change in the future, but now… As it is designed now, it is not realistic… who works in a university has a huge clinical workload and struggles to balance both, sometimes even compromising the quality of their work…”
Insufficient funding and lack of resources	“I just want to emphasize that, at least concerning the IRCCS and similar institutions… Right now, clinical work is saving the national healthcare system because these institutes are full of people whom I call ghosts for the government because they do not know they exist, hired on research funds but doing tons of clinical work… And having no time for research.”
Bureaucratic obstacles and long ethics approval times	“And the regulations, which sometimes are very stringent, very redundant, and much more complicated than what would actually be necessary for good clinical practice and scientific and safety standards for patients and operators… Also due to bureaucracy.”

#### Excessively Long Waiting Lists and Clinical Workload

First, healthcare providers mentioned the pervasive challenges in providing appropriate care arising from a lack of dedicated spaces and resources in the public healthcare system, resulting in strict time schedules, leaving them with little time even for essential tasks or needs. The combination of insufficient personnel and a scarcity of specialised staff adds to the issue, creating a scenario where healthcare providers are under an overwhelming burden left with little, if no, time for meals or deserved rest, let alone attentive care to patients.

This strain is clearly evident in the protracted waiting lists for patients, underscoring a systemic struggle where healthcare providers find themselves stretched thin, both in terms of physical resources and time, adversely impacting the quality and timeliness of patient care. A participant expressed the issue in the following quote: *“Because we do not have enough personnel, there are not dedicated spaces… we are literally chasing the covering of the waiting lists that continue to grow disproportionately…”*.

#### Geographical and Regional Disparities in Healthcare Resources

As well as for the patients, healthcare providers highlighted numerous disparities related to resource allocation in different geographical locations and regions. The vast majority of healthcare providers reported unequal distribution across the Italian regions and areas of financial, structural and personnel resources and diagnostic and therapeutic tools. Several healthcare providers explained how these differences have a crucial impact on the patients’ survival. For example, one participant stated: “*The time it took for the medical reports to reach the centre… and everything… five months passed… of course, when the diagnosis arrived, the patient had been dead for a while… and it is unacceptable…”*

The limited availability of advanced and innovative treatment and diagnostic tools, as well as the lack of uniformity in cancer diagnosis, also requires the patients who can afford it to travel to the northern regions, adding further workload to healthcare providers and facilities that operate in those better-equipped regions. As reported by the majority of healthcare providers, geographic disparities, mostly regional, are also related to socio-economic factors influencing screening awareness, additional delays due to obsolete equipment and to the fracture between allocated resources and the actual implementation in different regions. This hinders the possibility of having dedicated spaces and personnel that are adequate for the patients in need of care.

#### The Need for Greater Involvement of Psycho-Oncologists

During the focus groups, healthcare providers emphasised a critical challenge in the limited availability of psycho-oncologists who are specialised in addressing the psychological needs of cancer patients. This scarcity results from a simultaneous lack of specific allocation of resources and dedicated spaces. Another relevant sub-theme that emerged, regarding psychological support to cancer patients, is the indispensable role of psycho-oncologists in facilitating providers’ communication with patients and contributing to shared decision-making processes that are transparent and informed. Psycho-oncological support not only facilitates healthcare providers in managing these challenges but also enhances their ability to engage in empathetic and effective conversations with patients. Participants also emphasised the value of having a psycho-oncologist as an integral part of a multidisciplinary team, emphasising the importance of their constant presence for a better and more comprehensive clinical practice. However, transcripts also highlighted challenges associated with patients’ acceptance of psychological support. Despite the recognised significance of psycho-oncologists, some patients refuse this support, complicating scenarios for healthcare providers. The need for the constant presence of a psycho-oncologist and the patient’s resistance to accepting the support is articulated in the following quote: “*The most significant challenges arise in situations where family members recognise the need for psychological support, but patients refuse it*”.

#### Difficulty in Involving Patients in Shared Decision-making

Regarding shared decision-making processes, healthcare providers expressed a genuine desire to involve patients in decision-making, recognising its importance. However, the essence of oncological malignancies, which often necessitate prompt intervention, poses significant time constraints. Providers also acknowledged that clinical protocols, especially in well-studied or less severe situations, might limit the extent to which patients are involved in decision-making. Conversely, in cases of rare tumours where evidence-based interventions are less common, patients are more actively engaged in the decision-making process. The complexity arises from balancing established protocols with individualised choices, as illustrated in the following quote. “*It often happens to us to actively involve the patient in the decisions, especially with rare tumours for which we might not have extensive scientific data or numerous studies*”.

Another issue for healthcare providers is instances where patient involvement in decision-making is not possible due to a lack of the time and resources necessary to inform the patient regarding all the possibilities, the consequences and the possible impactful side effects; the patients cannot possibly make decisions if they are not properly informed.

#### The Need for Patient Empowerment

Healthcare providers agreed that the main goal, when speaking of disparities in accessing cancer care, would be that of empowering patients enhancing their awareness regarding prevention, booking procedures, access to treatment programs, lifestyle choices, but, mostly, with regards to their rights as oncological patients. As a matter of fact, issues arise when general practitioners do not inform the patients sufficiently or, as reported by the majority of the healthcare providers, when they are not fully aware of the procedures and management of oncological patients themselves: *“The general practitioner is the weak link... they are very overworked and are then the problem.”*

Lastly, healthcare providers highlighted how in some regions, such as Piedmont and Sardinia, there is a lack of patients’ associations and organisations which are usually of great help both to patients and healthcare providers. At least half of the healthcare providers emphasised the crucial importance of these associations in providing support and information to patients and, thus, lightening the workload of healthcare providers. Patients’ associations are also helpful in building networks between professionals on a regional and national level and providing help right within the centres or hospitals by both giving directions and informing patients and helping medical staff with administration and bureaucracy.

#### Limitations in the Integration of Clinical Practice and Research Activities

This last theme highlights the challenges faced by healthcare providers in integrating clinical practice and research activities, pointing to several structural complications. Healthcare providers express a genuine desire to engage in research, but excessive workloads often confine these activities to limited personal time, such as nights, free hours or even holidays. Despite a collective interest in research and a strong belief in its importance, time constraints, insufficient funding, and a lack of resources hinder the extent and quality of research efforts. Notably, a significant sub-theme emerges regarding clinicians being remunerated for research but primarily functioning in clinical roles, indicating a misalignment between roles and actual responsibilities. Indeed, a participant reported: “*Right now, clinical work is saving the National Healthcare System because these institutes are full of people whom I call ghosts for the government because they do not know they exist, hired on research funds but doing tons of clinical work*”. Bureaucratic obstacles and very slow ethics approval processes further complicate research endeavours, prolonging timelines and additionally burdening the workload.

## Discussion

In the current study, diverse cancer patients’ and healthcare providers’ characteristics served as a foundation for an exploration of reasons for disparities in cancer experiences in Italy. Indeed, different barriers and unmet needs possibly contributing to cancer disparities emerged during the online focus group discussions with patients and healthcare providers.

Overall, the identified themes, despite the specificities and uniqueness of the two groups (ie, patients and healthcare providers), show a high degree of consistency and similarity. These themes often complement each other, presenting two different facets or viewpoints of the same complex phenomenon. In particular, barriers related to hospitals, including *waiting lists*, *bureaucracy*, and *geographical and regional disparities*, were frequently reported by all participants. Both patients and healthcare providers expressed concerns about waiting lists and the unequal distribution of resources in hospitals. While patients were found to face the threat of prolonged waiting times, risking delayed treatment and potential compromise to both their survival and mental well-being, healthcare providers were found to navigate an overwhelming workload due to resource shortages. This challenges the providers’ capacity to deliver attentive and timely interventions increasing the patient load and, consequently, lengthening the waiting times for patients. This finding aligns with existing literature, as numerous studies conducted in Italy have highlighted the recurrent issue of excessively long waiting lists in accessing care through the public national healthcare system and how it impacts survival and quality of life.^30^ Evident differences in quality and availability of health services arise between regions and a clear fracture is observed between the north and the south of Italy.^31^

Furthermore, in both groups, the value of *psycho-oncological support* emerged. This highlights the recognition of the importance of addressing not only the physical aspects of cancer but also the psychological and emotional burden that comes with a cancer diagnosis. The value attributed to psycho-oncological support by cancer patients in the current study extends beyond the immediate challenges of treatment and recovery. It recognises the long-term psychological effects that a cancer diagnosis can have, emphasising the importance of ongoing support throughout the entire cancer care continuum. Addressing the emotional aspects of the cancer experience becomes essential to promoting resilience, coping mechanisms, and improved overall quality of life for individuals affected by cancer.^32^ However, as emerged from our results, the need for psycho-oncological support among cancer patients is often unmet, thus giving rise to disparities between those who have the opportunity to access such services due to awareness or referral, and those who are unaware of the existence of these services in hospitals. This discrepancy underscores a crucial aspect of healthcare inequality, where the accessibility of psycho-oncological support services may be contingent upon factors such as economic availability, information dissemination and referral pathways.^33^ In a recent publication,^20^ the lack of awareness regarding the benefits of psychological support in cancer patients, and its positive impact on survival and quality of life, hinders both the utilisation of such support by patients and the referral by healthcare providers. In addition, personal characteristics of the patients, as well as those of the healthcare providers, together with socio-economic factors, were found to prevent the access, the use and the provision of psycho-oncological support.^34^ On the side of healthcare providers, it became apparent from our results that there is a need for increased awareness and communication regarding psycho-oncological support services. Some patients may not be aware of these services due to a lack of information from healthcare professionals or a gap in communication channels.^35^ This points to an opportunity for healthcare providers to play a crucial role in bridging this information gap, ensuring that all individuals, irrespective of their background or level of knowledge, have equal access to valuable psycho-oncological support that can significantly impact their cancer journey. Moreover, in our findings, psycho-oncological support not only proves invaluable for the patients but also serves as a crucial resource for supporting healthcare providers. These support initiatives might act as a means to equip healthcare providers with the necessary tools to navigate the intricate emotional landscape of cancer.^36^

Patients’ *information needs* diverge from those of healthcare providers, yet they are complementary. On one hand, some patients have highlighted their frequent experience of encountering a lack of clear and comprehensive information and poor communication from healthcare providers. This perceived lack of clarity from healthcare providers aligns with the time constraints and stress often reported by providers due to overwhelming workloads, potentially impacting the depth of information provided to patients. In this case, the Internet and online resources could potentially address this misalignment between patients feeling under informed and healthcare providers lacking sufficient time to dedicate to patient informational support. Indeed, the Internet has proven advantageous for both patients and healthcare providers. Healthcare providers recognise the Internet’s value in empowering patients however, they highlight challenges associated with digitalisation, both within populations and hospital systems. Despite the Internet’s potential as a useful tool in overcoming certain barriers, as discussed in a recent publication, it may inadvertently give rise to new obstacles such as the potentially overwhelming abundance of information and the need to distinguish between reliable and untrustworthy sources.^18^ For more detailed information see Table 4.

Regarding *decision-making processes*, patients and healthcare providers shared slightly different yet complementary experiences. In these processes, patients express a desire for increased involvement in managing their illness and gaining a deeper understanding of its impact on their lives. On the other hand, healthcare providers acknowledge the constraints of protocols but aspire to enhance patient inclusion in medical decisions. Recent research highlights the numerous advantages of shared decision-making and active patient involvement in the care process.^37,38^ In this context, evidence in narrative medicine is noteworthy. This field emphasises the significance of patient and provider experiences through storytelling. Several studies have explored the feasibility and impact of narrative medicine in oncological clinical practice. For example, a digital platform has been developed to elicit guided narratives from patients undergoing chemotherapy or radiotherapy for solid tumours.^39^ This innovative approach aims to capture the human side of medicine, incorporating the experiences and reflections of both patients and providers.^40^

Lastly, themes that emerged only by patients’ results corresponded to *financial burden* and *interactions between patients and healthcare providers.* From the patients’ perspective, the economic burden associated with their oncological diagnosis and treatments was mainly represented by travel-related costs and expenses of private alternatives to public healthcare. This finding aligns with recent data indicating that approximately half of cancer patients experience personal economic challenges tied to the disease and its treatment, a phenomenon referred to as financial toxicity.^41^ Patients also reported the issue of ineffective and unsupportive interactions with their healthcare providers. They shared the need to experience empathetic and open communication with their physicians and the desire to be guided with adequate information on bureaucracy and patients’ rights by their oncologist and general practitioner. On the other side, a theme that only emerged from healthcare providers' results corresponded to the *difficulty in integrating clinical practice and research activities*. Despite the existing interest and desire to conduct research, practical obstacles such as insufficient time and resources strongly affect the extent and quality of the research endeavours.

The current study has important practical implications. First, the significance of psycho-oncological support highlights the necessity for inclusive and integrated healthcare strategies that address both the medical and psychosocial aspects of care. This approach should be accessible to a wider population, as emphasised in Germany’s National Cancer Plan, which aims to offer psycho-oncological care to all cancer patients based on their individual healthcare needs.^42^ Addressing such disparities may entail improving communication strategies within healthcare settings, implementing comprehensive training programs for healthcare providers to recognise and address the psychological needs of cancer patients, and establishing more accessible channels for patients to receive information about available support services, such as disseminating and implementing the European Society for Medical Oncology (ESMO) guidelines in many different healthcare systems.^43^

Secondly, since there has been an identified need for information, primarily from patients but also indirectly from healthcare providers, it would be beneficial to find a way to disseminate information in a simple and clear manner and provide comprehensive informational materials. The Internet could be advantageous in this regard. Despite the limitations and barriers to Internet access,^18^ the dissemination of reliable information online could help mitigate geographical disparities, facilitate access to essential resources, and foster a more informed healthcare community. It is crucial to explore effective strategies to ensure that information reaches both patients and healthcare professionals, promoting transparency and understanding in the healthcare communication landscape. The BEACON project, of which this study is a part, is oriented in this direction with the goal of centralising oncological information and making it publicly available. The objective is to empower stakeholders in the field of cancer, including patients, healthcare providers, researchers, and policymakers, by providing an online decision support tool with critical resources for cancer care and research.^44^

The strengths of this study include its multiple perspectives, with our sample involving not only patients but also healthcare providers. Moreover, another strength is the use of the COREQ guidelines to conduct and correctly report the methodology of the current study.^45^ Finally, considering the study’s aim to explore the possible factors contributing to cancer disparities, having had the opportunity to include participants from diverse regions, each with distinct clinical and personal histories and backgrounds, can be viewed as an added value that enhances our qualitative investigation.

The results of the study should be interpreted in light of potential limitations. One limitation is the small sample size of our study and the exclusion of a segment of the population, either elderly or those lacking access to devices or Internet connectivity.^46^ Moreover, the study is not longitudinal, and such temporal constraint should be considered in interpreting the findings because changes in participants’ perceptions and needs might emerge over time. Accordingly, other reasons for disparities might emerge as well as fluctuate along with participants’ experiences. Additionally, there is a prevalence of breast cancer patients in our sample possibly limiting our exploration of diversity of other cancer diagnoses. Similarly, the majority of healthcare providers operate in urban centres limiting the observation of healthcare dynamics in more remote areas. Despite the effort to implement a sampling strategy as inclusive as possible, the generalisability of the results may be limited by the fact that the participants who accepted are those who are already interested and sensitive to the topic.^47^ In terms of future directions, expanding the scope to include more countries and diverse healthcare systems could provide a broader understanding of cancer disparities. Additionally, incorporating longitudinal studies would enable a more dynamic exploration of the factors influencing cancer outcomes over time.

## Conclusion

Overall, the findings contribute valuable insights to inform inclusive healthcare strategies and address disparities in cancer care. The study underscores the persistent issue of excessively long waiting lists and regional disparities in accessing cancer care, aligning with existing literature highlighting its impact on survival and quality of life. The value of psycho-oncological support is emphasised, but disparities in access reveal the need for improved awareness and communication. The study also highlights the potential of the Internet in addressing information needs, despite associated challenges. Future directions involve expanding the study’s scope to include more countries and healthcare systems and incorporating longitudinal studies to better understand the evolving dynamics of cancer disparities over time.

## Supplemental Material

Supplemental Material - Understanding Reasons for Cancer Disparities in Italy: A Qualitative Study of Barriers and Needs of Cancer Patients and Healthcare Providers Supplemental Material for Understanding Reasons for Cancer Disparities in Italy: A Qualitative Study of Barriers and Needs of Cancer Patients and Healthcare Providers by Giulia Ferraris, Veronica Coppini, Maria V. Ferrari, Dario Monzani, Roberto Grasso, and Gabriella Pravettoni in Cancer Control

Supplemental Material - Understanding Reasons for Cancer Disparities in Italy: A Qualitative Study of Barriers and Needs of Cancer Patients and Healthcare Providers Supplemental Material for Understanding Reasons for Cancer Disparities in Italy: A Qualitative Study of Barriers and Needs of Cancer Patients and Healthcare Providers by Giulia Ferraris, Veronica Coppini, Maria V. Ferrari, Dario Monzani, Roberto Grasso, and Gabriella Pravettoni in Cancer Control

## Data Availability

The datasets used and/or analysed during the current study are available from the corresponding author on reasonable request.*

## References

[1] Chandra PS Chaturvedi SK Channabasavanna SM , et al. Psychological well-being among cancer patients receiving radiotherapy – a prospective study. Qual Life Res. 1998;7(6):495-500. 9737139 10.1023/a:1008822307420

[2] Franzini L Giannoni M . Determinants of health disparities between Italian regions. BMC Publ Health. 2010;10(1):296. 10.1186/1471-2458-10-296 PMC2902435 20515482

[3] Vaccarella S Georges D Bray F , et al. Socioeconomic inequalities in cancer mortality between and within countries in Europe: A population-based study. Lancet Reg Health – Eur. 2022;25:100551. 36818237 10.1016/j.lanepe.2022.100551 PMC9929598

[4] Loerzel VW Bushy A . Interventions that address cancer health disparities in women. Fam Community Health. 2005;28(1):79-89. 15625508 10.1097/00003727-200501000-00010

[5] Collatuzzo G Ferrante M Ippolito A , et al. Cancer in migrants: A population-based study in Italy. Cancers Basel. 2023;15(12):3103. 37370713 10.3390/cancers15123103 PMC10295978

[6] National Cancer Institute. Cancer Disparities . National Cancer Institute. Accessed December, 2023. https://www.cancer.gov/about-cancer/understanding/disparities

[7] Afshar N English DR Milne RL . Factors explaining socio-economic inequalities in cancer survival: A systematic review. Cancer Control. 2021;28:10732748211011956. 33929888 10.1177/10732748211011956 PMC8204531

[8] Giorgi Rossi P Djuric O Navarra S , et al. Geographic inequalities in breast cancer in Italy: Trend analysis of mortality and risk factors. Int J Environ Res Publ Health. 2020;17(11):4165. 10.3390/ijerph17114165 PMC7312287 32545263

[9] Gehlert S Hudson D Sacks T . A critical Theoretical approach to cancer disparities: Breast cancer and the social determinants of health. Front Public Health. 2021;9:674736. 34095075 10.3389/fpubh.2021.674736 PMC8175790

[10] Mauro M Giancotti M . The 2022 primary care reform in Italy: Improving continuity and reducing regional disparities? Health Pol. 2023;135:104862. 10.1016/j.healthpol.2023.104862 37399680

[11] Hyatt A Shelly A Cox R Humphries E Lock G Varlow M . How can we improve information for people affected by cancer? A national survey exploring gaps in current information provision, and challenges with accessing cancer information online. Patient Educ Counsel. 2022;105(8):2763-2770. 10.1016/j.pec.2022.04.009 35465976

[12] Thiessen M Sinclair S Tang PA Bouchal SR . Information access and use by patients with cancer and their friends and family: Development of a Grounded theory. J Med Internet Res. 2020;22(10):e20510. 33118940 10.2196/20510 PMC7661235

[13] Boons CCLM Harbers L Timmers L , et al. Needs for information and reasons for (non)adherence in chronic myeloid leukaemia: Be aware of social activities disturbing daily routines. Eur J Haematol. 2018;101(5):643-653. 10.1111/ejh.13155 30058149

[14] Ferrari M Ripamonti CI Hulbert-Williams NJ Miccinesi G . Relationships between unmet needs, depression and anxiety in non-advanced cancer patients. Tumori. 2019;105(2):144-150. 29714666 10.1177/0300891618765546

[15] Clarke MA Moore JL Steege LM , et al. Health information needs, sources, and barriers of primary care patients to achieve patient-centered care: A literature review. Health Inf J. 2016;22(4):992-1016. 10.1177/1460458215602939 26377952

[16] Ramsey I Corsini N Peters MDJ Eckert M . A rapid review of consumer health information needs and preferences. Patient Educ Counsel. 2017;100(9):1634-1642. 10.1016/j.pec.2017.04.005 28442155

[17] Williams MV Davis T Parker RM Weiss BD . The role of health literacy in patient-physician communication. Fam Med. 2002;34(5):383-389. 12038721

[18] Ferraris G Monzani D Coppini V , et al. Barriers to and facilitators of online health information-seeking behaviours among cancer patients: A systematic review. Digit Health. 2023;9:20552076231210663. 38107979 10.1177/20552076231210663 PMC10725105

[19] Mehnert-Theuerkauf A Hufeld JM Esser P , et al. Prevalence of mental disorders, psychosocial distress, and perceived need for psychosocial support in cancer patients and their relatives stratified by biopsychosocial factors: rationale, study design, and methods of a prospective multi-center observational cohort study (LUPE study). Front Psychol. 2023;14:1125545. 37151329 10.3389/fpsyg.2023.1125545 PMC10157044

[20] Coppini V Ferraris G Monzani D Grasso R Pravettoni G . Disparities and barriers in the assessment of psychological distress, access to and use of psycho-oncological support in Europe: current perspectives. Front Psychol. 2023;14:1252843. 37794912 10.3389/fpsyg.2023.1252843 PMC10546339

[21] Lion KM Pike KE Dhillon HM , et al. Access to psychosocial support for people with brain tumor and family members: Healthcare professional perspectives. Psycho Oncol. 2023;32(6):980-988. 10.1002/pon.6142 37084182

[22] European Cancer Information System . European Commission. Accessed December, 2023. https://ecis.jrc.ec.europa.eu/

[23] Istituto Superiore diTumori Sanità. : nel 2022 in Italia stimati 390.700 nuovi casi, circa 14 mila in più in 2 anni. Istituto Superiore di Sanità. Accessed December, 2023. https://www.iss.it/-/tumori-nel-2022-in-italia-stimati-390.700-nuovi-casi-circa-14-mila-in-pi%C3%B9-in-2-anni

[24] OECD . EU Country Cancer Profile: Italy 2023 [Internet]. Paris: Organisation for Economic Co-operation and Development; 2023. https://www.oecd-ilibrary.org/social-issues-migration-health/eu-country-cancer-profile-italy-2023_a0a66c1d-en;jsessionid=7RzFr2TZDdd45WTN5zGxdb4jNv9kjCLWpP_djBgB.ip-10-240-5-82

[25] Mezzetti M Palli D Dominici F . Combining individual and aggregated data to investigate the role of socioeconomic disparities on cancer burden in Italy. Stat Med. 2020;39(1):26-44. 31746020 10.1002/sim.8392 PMC7939453

[26] Tong A Sainsbury P Craig J . Consolidated criteria for reporting qualitative research (COREQ): a 32-item checklist for interviews and focus groups. Int J Qual Health Care. 2007;19(6):349-357. 17872937 10.1093/intqhc/mzm042

[27] Ferraris G Coppini V Monzani D , et al. Addressing disparities in European cancer outcomes: a qualitative study protocol of the BEACON project. Front Psychol. 2024;15. 10.3389/fpsyg.2024.1252832 PMC10925749 38469221

[28] Guest G Bunce A Johnson L . How many interviews are enough? An experiment with data saturation and variability. Field Methods. 2006;18(1):59-82.

[29] Braun V Clarke V . Successful Qualitative Research : A Practical Guide for Beginners [Internet]. 1st ed. Sage Publications Ltd; 2013. 400 p. Available from: https://www.torrossa.com/en/resources/an/5017629

[30] Matranga D Maniscalco L . Inequality in healthcare utilization in Italy: How important are barriers to access? Int J Environ Res Publ Health. 2022;19(3):1697. 10.3390/ijerph19031697 PMC8835011 35162720

[31] De Rosis S Guidotti E Zuccarino S Venturi G Ferré F . Waiting time information in the Italian NHS: A citizen perspective. Health Pol. 2020;124(8):796-804. 10.1016/j.healthpol.2020.05.012 32624247

[32] Tran TXM Jung SY Lee EG , et al. Fear of cancer recurrence and its negative impact on health-related quality of life in long-term breast cancer survivors. Cancer Res Treat. 2022;54(4):1065-1073. 34883553 10.4143/crt.2021.835 PMC9582487

[33] Hecht K Günther MP Kirchebner J , et al. Predictive factors associated with declining psycho-oncological support in patients with cancer. Curr Oncol. 2023;30(11):9746-9759. 37999127 10.3390/curroncol30110707 PMC10670809

[34] Günther MP Schulze JB Kirchebner J Jordan KD von Känel R Euler S . Severe mental illness in cancer is associated with disparities in psycho-oncological support. Curr Probl Cancer. 2022;46(3):100849. 35325803 10.1016/j.currproblcancer.2022.100849

[35] Alemu W Girma E Mulugeta T . Patient awareness and role in attaining healthcare quality: a qualitative, exploratory study. Int J Afr Nurs Sci. 2021;14(5):100278.

[36] Aldaz BE Treharne GJ Knight RG Conner TS Perez D . Oncology healthcare professionals’ perspectives on the psychosocial support needs of cancer patients during oncology treatment. J Health Psychol. 2017;22(10):1332-1344. 26837692 10.1177/1359105315626999

[37] Chichua M Brivio E Mazzoni D Pravettoni G . Shared decision-making and the lessons learned about decision regret in cancer patients. Support Care Cancer. 2022;30(6):4587-4590. 35031827 10.1007/s00520-021-06725-5 PMC9046326

[38] Savioni L Triberti S Durosini I Pravettoni G . How to make big decisions: A cross-sectional study on the decision making process in life choices. Curr Psychol. 2022;42(18):15223-15236.

[39] Cercato MC Colella E Fabi A , et al. Narrative medicine: feasibility of a digital narrative diary application in oncology. J Int Med Res. 2022;50(2):03000605211045507. 35107030 10.1177/03000605211045507 PMC8859529

[40] Hinyard LJ Wallace C Trees A Ohs J . Narrative medicine: A useful approach for difficult conversations. J Clin Oncol. 2019;37(31_suppl):6.

[41] Smith GL Banegas MP Acquati C , et al. Navigating financial toxicity in patients with cancer: A multidisciplinary management approach. CA A Cancer J Clin. 2022;72(5):437-453. 10.3322/caac.21730 PMC12994614 35584404

[42] Kusch M Labouvie H Schiewer V , et al. Integrated, cross-sectoral psycho-oncology (isPO): a new form of care for newly diagnosed cancer patients in Germany. BMC Health Serv Res. 2022;22(1):543. 35459202 10.1186/s12913-022-07782-0 PMC9034572

[43] European Society for Medical Oncology. Guidelines by Topic. ESMO. Accessed December, 2023. https://www.esmo.org/guidelines/guidelines-by-topic

[44] European Commission . BEACON - Cancer Care Beacon - Reducing Disparities Across the European Union. European Commission. Accessed December, 2023. https://health.ec.europa.eu/non-communicable-diseases/cancer/europes-beating-cancer-plan-eu4health-financed-projects/projects/beacon_en

[45] O’Brien BC Harris IB Beckman TJ Reed DA Cook DA . Standards for reporting qualitative research: a synthesis of recommendations. Acad Med. 2014;89(9):1245-1251. 24979285 10.1097/ACM.0000000000000388

[46] Hunsaker A Hargittai E . A review of Internet use among older adults. New Media Soc. 2018;20(10):3937-3954.

[47] Robinson OC . Sampling in interview-based qualitative research: A theoretical and practical guide. Qual Res Psychol. 2014;11(1):25-41.

