# Supplementary material for: Understanding Reasons for Cancer Disparities in Italy: A Qualitative Study of Barriers and Needs of Cancer Patients and Healthcare Providers
Source: Cancer Control. 2024 Jun 19;31:10732748241258589. doi: 10.1177/10732748241258589 (PMC11189013; doi:10.1177/10732748241258589)
Supplement: Supplemental Material - Understanding Reasons for Cancer Disparities in Italy: A Qualitative Study of Barriers and Needs of Cancer Patients and Healthcare Providers [file sj-pdf-2-ccx-10.1177_10732748241258589.pdf]

Additional file 2

Professional role and expertise of Italian Healthcare Providers

Professional role and expertise	Healthcare Providers	
	n	%
**Occupational Role**		
Psycho-oncologist	4	26.7
Surgical oncologist	2	
Medical oncologist	8	62.5
Radiation oncologist	2	13.3
**Working experience in the oncological field**		
0-3	0	0
3-6	2	13.3
6-9	2	13.3
> 9	12	75
**Number of Hospitals or Centres**		
1-2	10	66.7
2-4	6	40
4-6	0	0
6+	0	0
**Other Hospitals or Centres Abroad**		
No	7	46.7
United Kingdom	3	20
Sweden	2	13.3
Latin America	1	6.7
USA	2	13.3
France	2	13.3
Netherlands	1	6.7
Spain	1	6.7
**Type of treated cancer**		
Neuroendocrine Tumour	1	6.7
Various/multiple types	6	40
Sarcomas	3	20
Gastrointestinal	3	20
Breast	1	6.7
Prostate	1	6.7
Head and Neck	1	6.7
**Research work**		
No	2	13.3
Yes	14	87.5
